# Co‐designing clinical trials alongside youth with chronic pain

**DOI:** 10.1002/pne2.12105

**Published:** 2023-05-08

**Authors:** Zina Zaslawski, Katherine Dib, Vivian W. L. Tsang, Serena L. Orr, Kathryn A. Birnie, Trinity Lowthian, Zahra Alidina, Melila Chesick‐Gordis, Lauren E. Kelly

**Affiliations:** ^1^ George and Fay Yee Centre for Healthcare Innovation Winnipeg Manitoba Canada; ^2^ Children's Hospital Research Institute of Manitoba Winnipeg Manitoba Canada; ^3^ Patient Partner Canadian Collaborative for Childhood Cannabinoid Therapeutics (C4T) Halifax Nova Scotia Canada; ^4^ KidsCan Young Persons' Research Advisory Group under the Maternal Infant Child and Youth Research Network Vancouver British Columbia Canada; ^5^ Division of Neurology Alberta Children's Hospital Calgary Alberta Canada; ^6^ Department of Pediatrics, Cumming School of Medicine University of Calgary Calgary Alberta Canada; ^7^ Department of Anesthesiology, Perioperative, and Pain Medicine University of Calgary Calgary Alberta Canada; ^8^ Department of Community Health Sciences University of Calgary Calgary Alberta Canada; ^9^ Patient Partner Canadian Collaborative for Childhood Cannabinoid Therapeutics (C4T) Ottawa Ontario Canada; ^10^ Patient Partner Canadian Collaborative for Childhood Cannabinoid Therapeutics (C4T) Holland Landing Ontario Canada; ^11^ Patient Partner Canadian Collaborative for Childhood Cannabinoid Therapeutics (C4T) Vancouver British Columbia Canada; ^12^ Department of Pharmacology and Therapeutics, Max Rady College of Medicine University of Manitoba Winnipeg Manitoba Canada

**Keywords:** clinical trials, headache, pain, patient engagement, pediatrics

## Abstract

Youth have a right to participate in research that will inform the care that they receive. Engagement with children and young people has been shown to improve rates of enrollment and retention in clinical trials as well as reduce research waste. The aim of the study is to gain practical insight on the design of trials specifically on (1) recruitment and retention preferences, (2) potential barriers to research, and (3) study design optimization. Based on this youth engagement, we will co‐design two clinical trials in headaches with youth. Two recruitment strategies were used to recruit 16 youth from across Canada (aged 15–18 years) from an existing youth group, the KidsCan Young Persons' Research Advisory Group (YPRAG) and a new youth group in collaboration with Solutions for Kids in Pain (SKIP). Four virtual, semi‐structured discussion groups were held between April and December 2020, which included pre‐circulated materials and utilized two distinct upcoming planned trials as examples for specific methods feedback. Individual engagement evaluations were completed following the final group session using the Public and Patient Engagement Evaluation Tool. Descriptive results were shared with participants prior to publication to ensure appropriate interpretation. The discussion was centred around three themes: recruitment and retention preferences, potential barriers to participation, and study design optimization. Youth indicated that they would prefer to be contacted for a potential study directly by their physician (not over social media), that they would like to develop rapport with study staff, and that one of the barriers to participation is the time commitment. The youth also provided feedback on the design of the clinical trial including outcome measurement tools, data collection, and engagement methods. Feedback on the virtual format of the engagement events indicated that participants appreciated the ease of the online discussion and that the open‐ended discussion allowed for easy exchange of ideas. They felt that despite a gender imbalance (towards females) it was an overall inclusive environment. All participants reported believing that their engagement will make a difference to the work of the research team in designing the clinical trials. Perspectives from a diverse group of youth meaningfully improved the design and conduct of two clinical trials for headaches in children. This study provides a framework for future researchers to engage youth in the co‐design of clinical trials using online engagement sessions.

## 1 INTRODUCTION

Engaging with children and youth in all stages of research is valuable to designing meaningful studies that promote evidence‐based practices and therapeutics. Research has shown that involving youth in their medical decisions increases adherence to treatment and improves overall health outcomes.^1^ As such, the World Health Organization recommends actively involving youth as collaborators in all stages of research, from design to evaluation.^2^, ^3^, ^4^ Involving youth in the design of clinical trials reflects a culture change towards patient‐oriented research.

Canadian Institutes of Health Research (CIHR) defines patient partners as “Patients [who] become patient partners in the project and can be actively engaged in governance, priority setting, developing the research questions, and even performing certain parts of the research itself.”^5^ While it might seem obvious to include those who will ultimately benefit from the research in the design process, there is widespread lack of involvement of patient partners in the research process, especially in pediatric research.^6^, ^7^, ^8^ The downfall of limited or no patient engagement is that treatment guidelines^9^ are designed without practical and pragmatic input from end‐users, leading to research waste.^10^, ^11^ To reduce the potential for research waste, research teams should partner with youth and families early on in the study design process to determine research questions, identify research priorities, improve feasibility, and ensure meaningful outcomes.^12^ Young people can be involved in clinical research in many forms including as research advisors (also called patient partners), who partner with researchers and participate in decision making including the study design. A second way is for youth to participate in research studies that are designed to learn about ways to better design research, such as the study we report on below.

Children and adolescents have a right to participate in research that will inform their care. Many therapies are still not approved for children in pain, and the available evidence is often based on studies in adults.^13^ Pediatric‐specific trials will improve medical care,^13^ however, these studies can only answer questions if they are successful at recruiting and retaining participants. There are clear benefits when youth provide insight and expertise to the study protocol and research materials.^3^, ^4^, ^8^, ^12^, ^14^, ^15^ By co‐designing clinical trials with youth, recruitment tools (including consent and assent forms), and outcome measures will be more accessible^12^, ^16^ leading to increased comprehension and improved rates of enrollment and retention.^16^ Furthermore, when the trial is completed, youth collaborators can add context by jointly interpreting the results^16^ and planning strategies for knowledge translation. Having youth review the data can help to determine if the findings, conclusions, and recommendations reflect their perspective and needs.

The main goal of this project was to gain perspectives from youth in the co‐development of clinical trial designs related to chronic pain.^17^, ^18^ This project includes a novel collaboration between Maternal Infant Child Youth Research Network (MICYRN), the KidsCan Young Persons' Research Advisory Group (YPRAG), Solutions for Kids in Pain (SKIP), and the Canadian Collaborative for Childhood Cannabinoid Therapeutics (C4T) research consortium. The specific objectives of this research study include gaining insight from youth on (1) recruitment and retention preferences, (2) potential barriers to research, and (3) study design optimization in clinical trials involving children and adolescents with pain, specifically headaches.

## 2 METHODS

### 2.1 Sampling and recruitment

We (The project team‐ LEK, SLO, KB, ZZ, VWLT, KD), used two different approaches to recruitment for this study, first with an established group of youth who have experience co‐designing clinical trials, and second with a group of youth with lived experience with chronic pain. The KidsCan YPRAG is a group of 14 young people (ages 13–19 years) who have experienced chronic health conditions with diverse socioeconomic, geographic, and cultural backgrounds from across Canada. This youth advisory group is a national initiative aimed to increase the engagement of pediatric patients in all phases of the research process.^19^ We selected this group due to their familiarity with clinical trials, proximity to recruitment sites, and interest in participating in our study. In addition, we collaborated with Solutions for Kids in Pain (SKIP) to obtain insight directly from youth with chronic pain. SKIP is a national knowledge mobilization network on a mission to improve children's pain management with evidence‐based solutions through coordination and collaboration.^20^ We used convenience sampling to identify youth via SKIP's Patient Partner network who were aged 13–18 years and specifically had lived experience of headaches. We aimed to recruit youth from diverse socioeconomic, geographic, and cultural backgrounds. We created a 1‐page description of the engagement opportunity and inclusive graphic that was disseminated through social media (Twitter, Instagram, Facebook) and through email (see Appendix A). Youth were compensated $25 CAD/h for two 90‐min sessions.

### 2.2 Participants

Two pan‐Canadian groups of youth, (KidsCan YPRAG and via SKIP), were engaged in virtual discussion groups for this study. Two youth partners (VWLT, KD) were included in all planning and facilitation of the meetings. They also contributed to the production of this manuscript. Seven young people (four females and three males) from the KidsCan YPRAG attended the discussion groups from Ontario, Manitoba, Alberta, and British Columbia. Our recruitment thorough SKIP resulted in engagement with nine youth aged 15–18 years (eight females and one male) with lived experience of headaches from Nova Scotia, Ontario, Alberta, and British Columbia. See Table 1 for a description of self‐identification of youth recruited through SKIP.

**TABLE 1:** Self‐identification of youth recruited through SKIP.

Self‐identification of youth recruited through SKIP	
Variable	*n*
Racialized individuals	
Yes	2
No	7
Person with disability	
Yes	3
No	4
Indigenous	
Yes	0
No	9
Recent immigrants	
Yes	0
No	9
LGBTQ	
Yes	2
No	7
Duration of headaches/migraines	
1–2 years	2
3–4 years	5
5 years	1
8 years	1

### 2.3 Informed consent process

Although research ethics board approval is not a requirement for the overall engagement of patients and the public, our goal was to conduct a specific research study to gain perspectives on clinical trial designs and share youth perspectives. As such, we sought prospective research ethics approval from the University of Manitoba Health Research Ethics Board so our learnings from youth could be published and applied by researchers in other areas.

Two weeks prior their first meeting, youth were provided with consent forms along with a brief introduction and goals for the discussion groups. Consent forms were signed by the youth and their parent/guardian (if they were under 18) and returned to the research team by email. Youth and their parents were encouraged to contact the primary investigator or study staff with any questions. All participants were able to withdraw their consent during the study for any reason.

### 2.4 Clinical trial examples

Two upcoming clinical trials led by LEK and SLO were described to youth and used for specific discussion questions. These studies include (1) a tolerability study of a CANnabinoids for Chronic Headaches in Adolescents (CAN‐CHA, more information available on ClinicalTrials.gov NCT05337033), and (2) a clinical trial exploring non‐IV treatment for migraine attacks in the emergency department.

#### 2.4.1 Study 1

The CAN‐CHA clinical trial is an academic‐led trial funded by the Canadian Institutes of Health Research and the SickKids Foundation and led by C4T (LEK). This multi‐site study will recruit 20 adolescents with chronic migraine to a dose escalation, open label, tolerability trial investigating the safety of a cannabidiol‐enriched cannabis product.

#### 2.4.2 Study 2

The non‐IV treatment for migraine attacks in the emergency department study is a clinical trial funded by the Canadian Institutes of Health Research (SLO). This single site, pilot, double‐dummy, randomized controlled trial will recruit 40 youth aged 8–18 years visiting the emergency department with migraine attacks with the aim of determining the feasibility of comparing the standard of care IV interventions to a novel neuromodulation device for the treatment of migraine attacks.

### 2.5 Discussion group format

We facilitated four discussion groups over an video teleconferencing online software program‐Zoom^21^; two with YPRAG in April and September of 2020, and two with youth with chronic pain (via SKIP) in December 2020. Youth partners (VWLT and KD) did not contribute data to the discussion groups but were present for facilitation purposes. There were three main areas where youth expertise was sought: recruitment and retention preferences, barriers to research participation in clinical trials, and study design optimization. We held the virtual discussion groups to identify research questions and outcomes that are important to the group, for example, quality of life, sleep, and school performance. We discussed the research processes (recruitment, data collection, dissemination and implementation) for Study 1 (the dose finding study). In addition, we asked the group about their preferences for a non‐IV intervention for the treatment of migraine attacks in the emergency department (Study 2).

The meetings started with introductions of the research team and the youth. Everyone shared their name, the city they are from, how long they lived with headaches, and a hobby they might have picked up during the COVID‐19 lockdown. Following the introductions, the primary investigators (LEK, SLO) shared specific information about the studies in question using PowerPoint and answered questions arising from the youth. The chair of YPRAG (VWLT), who has led the group for the past several years, facilitated the meeting with YPRAG. The meetings with youth with chronic pain were co‐led by a youth partner (KD), the knowledge broker for C4T (ZZ), and the PI of one of the studies (SLO). Consent for recording the meetings to ensure appropriate interpretation was provided and documented in the informed consent documents. Each meeting focused on a different study although the discussions were not exclusively study specific.

*Study 1: A dose finding study evaluating a cannabinoids for chronic headaches in adolescents (CAN‐CHA)*.

The discussions were centered on five main open‐ended questions:

1. What outcomes are important to you for us to study and measure? Physical, mental and social health factors that might relate to chronic daily headaches.
2. What is the best way to reach youth with chronic daily headaches to participate in research?
3. What are potential barriers for participation?
4. What are the best methods to encourage study retention?
5. What could be done to make it easier to participate in the study? This includes any strategies for pain management.

Youth were presented a draft of a daily diary (see Appendix B) proposed for study #1 and were asked to comment on the following:

1. Would you complete this diary every day?
2. Would you stay committed to completing every day?
3. Would you prefer to do this 1 week a month or every day?
4. What would help you remember to complete it?
5. How would you prefer to complete this? Computer, phone, other.

For the last portion of the meeting, we asked the youth how often they would like to fill out various outcome measurement tools and if the tools selected were accessible, logical, and meaningful. Youth were presented with a number of short, validated questionnaires about pain interference, sleep interference, and mental health (positive affect, depression, and anxiety). As invited by the youth, additional discussion groups will be held once study results are available, for the purposes of soliciting interpretation by the youth and to plan knowledge translation and education strategies. In addition, youth with chronic pain who participated in the engagement activities were invited to engage further with the study team, if interested.

*Study 2: A clinical trial looking at non‐IV treatment for migraine attacks in the emergency department*.

For the second study, the aim of the meeting was to co‐select an intervention for a pilot randomized controlled trial where standard of care IV interventions will be compared to a novel, non‐IV intervention for treating attacks of migraine. During this meeting, one multiple‐choice question, using the poll function in Zoom, and open‐ended questions were used to facilitate the discussion. After a PowerPoint presentation of the four potential non‐IV interventions to treat acute attacks of migraine in the emergency department, youth were asked to vote on “which treatment would you choose to treat a migraine attack in the emergency department?”

The open‐ended questions were asked as follow‐up to the four potential treatments to inform the design of future research. Some of the questions were:

1. What do you think of the different devices? What do you think the positives and negatives are about each device?
2. Do you think we should design a study where we test one of these devices, or a study where patients are offered the option of choosing which treatment they would like to receive?
3. Do you have any specific ideas about how we can design this study to make it better for patients?
4. If you were to receive one of the treatments in the emergency department, would you want to receive it before, during, or after the standard intravenous treatment?
5. Is there anything in particular you think the researchers should consider when making plans for a study on how to treat kids and teens who go to the emergency department for migraine?
6. What would make you more likely to participate in a study on treating kids and teens with migraine in the emergency department?

### 2.6 Analysis plan

**FIGURE 1:**
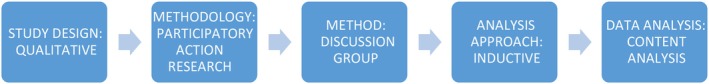
Study design overview.

Following all engagement sessions, the recordings were transcribed for analysis. Content analysis was selected as the analytical method, as it is a descriptive approach that entails literal interpretation of the data into themes.^22^ See Figure 1 for study design overview.

For study 1: the CAN‐CHA study, an action plan was created. To organize the plan a table was created with what the researchers heard (i.e. the themes) and their action items (See Appendix C). Data from the youth were extracted and organized into themes of comments and suggestions. ZZ and LEK provided the action items based on the youth comments and suggestions. To increase the validity of our results, we conducted member checking. We shared the table (Appendix C) with the youth who participated in the discussion groups for feedback. There were no further changes made to the table following the participant validation.

For study 2: the non‐IV treatment for migraine attacks in the emergency department study, the results of youth voting were compiled and used to select a target intervention for the study, among four possible interventions presented during the engagement session. Other feedback regarding study design was summarized from the transcripts and used to optimize the study protocol. Since study 2 was focused on selecting the intervention to study, which was chosen during the session, no validation was completed for this objective.

### 2.7 Evaluation and reporting

Participants were sent a standardized questionnaire to evaluate the quality of the engagement sessions, and to provide an open‐ended opportunity to further comment on the clinical trial design. We used the PPEET (Public and Patient Engagement Evaluation Tool).^23^ created by researchers at McMaster University to evaluate and report on various patient and public engagement activities within health research. The PPEET consists of three questionnaires that, when used together, are meant to offer a complete evaluation of the patient and public engagement. Each of the three questionnaires is composed of multiple choice and open‐ended questions. For our purposes, we used Module A‐ One Time Engagement Activities. This module consists of 21 items organized into five parts: background information, communication and support for participants, sharing your views and perspectives, impact and influence of the engagement initiative and final thoughts. The strength of this tool is that it was designed for use by researchers, patient partners, and the organizations.^23^ Feedback was sent to the youth groups highlighting how the input from the groups was implemented in the research process. It has been stated that previous reporting of engagement activities have not sufficiently described the context, mechanism, and expected outcomes.^24^ To improve the quality, transparency, and consistency of reporting on patient and public involvement, we followed the guidelines presented in GRIPP‐2.^25^

## 3 RESULTS

### 3.1 Recruitment and retention preferences for clinical trials

We asked what young people think are the best means to contact potential research participants for clinical trials for pediatric chronic pain. Most suggested that they preferred to be contacted by their medical care team (e.g., their physician can bring up the study during an appointment and share information with potential participant and their parents). Some said they would be more likely to respond to a research study email or call if it is personalized rather than a generic invitation email. Most youth agreed that they would not respond to an ad on social media. One youth said that they have been taught to be cautious about what they see and read on social media, and others said they would be hesitant to follow a link to a research study if they see it is a targeted ad. One youth suggested posting on local hospital websites instead, which is viewed as a more credible source of information about research studies. This may be different depending on the type of ad (survey, engagement, clinical trial) being promoted on social media and the credibility of the source. Regarding the ease of participating in trials, we asked youth how we could make this easier and retain people who do agree to be in research studies. Some youth suggested calling the participants to check in if they miss several days of data collection. However, other youth said that they prefer emails or texts and not calls, as it is a written reminder for them to complete the tasks. The youth also had mixed opinions on how often to call, email, or text as reminders. Some said they prefer a daily text reminding them which research tasks to complete and some preferred a call only when they missed tasks several days in a row. Consensus was not reached on a preferred frequency of follow‐up.

Participants also reported individual pain management and fear management strategies that could help improve the comfort of blood draws, if needed as part of the clinical trial design. Reducing anxiety around blood draws and effective pain management was a factor for the participants when were asked about enrolling and staying in a study. Some youth mentioned that they prefer to be distracted while others mentioned they prefer to know exactly what is happening and to get it over with quickly. Having an individual conversation with the research nurse about their preferred evidence‐based pain and fear management strategies, and offering multiple coping techniques was suggested to ensure youth feel comfortable and safe for the duration of the research study while applying evidence‐based approaches to reducing blood draw pain. The youth also preferred the blood draw at the same place, same time, with the same nurse or someone they knew who was affiliated with the study each time and preferred not to have to travel to a different location. Another suggestion the youth made to ease participation is to have snacks available, especially during longer appointments and around lunch time.

### 3.2 Potential barriers (and solutions) to participation in clinical trials

Some youth presented time constraints as their main barrier to participation in research studies. This was especially important to youth who said they have very limited times where they are pain‐free. Others have also mentioned how time constraints may be a barrier for parents who might need to take time off work to drive them to appointments. To address these barriers, youth recommended that research appointments happen on the same days and times each month (e.g., the 15th at 3 PM) so that they could plan their school and social activities around those appointments and parents would know well in advance when time away from work would be needed. Lack of information about the study was another barrier to participation that the youth discussed, as well as very little health information for patients is specifically developed for very young children. Youth reported requesting full details of the study procedures/interventions in an accessible format before agreeing to participate in the study. One youth suggested creating an infographic and a video that explains the study and its impact as a recruitment tool.

### 3.3 Study design optimization

Participants were asked study specific questions for two upcoming clinical trials. When asked what important outcomes researchers need to consider in clinical trials for primary headache disorders, youth agreed that intensity and frequency of headaches are the most important outcomes. The discussion on pain also included mention of how intensity of pain is very subjective and even changes for the person depending on many factors. Some youth recalled that their physician is using the “traffic light” system to assess the intensity of headaches that was easy to complete. The traffic light system is designed to help patients describe the degree of disability associated with their headache or migraine attack. Green means that they can still go (i.e., function) with this headache, yellow means they have to slow down due to the headache, and red means they have to stop due to the headache.^26^ The traffic light system pertain to outpatient acute headache management plans however and is not designed to be used in the emergency department. Youth also mentioned the degree of impact of headaches on daily activities like school and extracurriculars as an important outcome. One suggested withdrawing from and canceling various activities would be way to measure this outcome. Recommendations for including pain, headache impact and functional (activity limiting) outcomes were highlighted by participants.

### 3.4 Study specific recommendations

#### 3.4.1 Study 1: A multisite tolerability study called CANnabinoids for Chronic Headaches in Adolescents (CAN‐CHA)

For the CAN‐CHA study, participants will be completing a daily diary online or on paper to track headaches, pain, and concomitant interventions such as psychotherapy or pain medications. The research team asked the youth how to ensure that the participants remain engaged and stay committed to completing the daily diaries over a 6‐month study period. One suggestion was to provide participants the option of visualizing their progress weekly or monthly. For example, the research team could send out weekly emails with charts on how they rated their pain each day or on the frequency and duration of headaches. Another suggestion was to set up a reward system, where participants receive something in return for completing a certain percentage of daily diaries within a timeframe. Another practical suggestion was to include daily text reminders with a link that would open the data collection tool.

Youth were provided with three options for diary data collection frequency: every day during the study, in bursts (every day for 1 week each month), or when triggered by a headache. Most youth agreed that they would prefer to enter daily for the duration of the study so that it became a part of their routine. One youth said that when they tried to use an app to track headaches, they only completed it after a headache event so it did not accurately reflect their entire experience. Another youth suggested that daily data collection would result in a more accurate and a better representation of pattern of headaches. The research team originally planned to collect outcome measures on pain interference, sleep disturbance, and mental health once in the beginning and once at the end. Youth suggested adding another data collection point the middle for a better representation of results, and all agreed that completing these questionnaires monthly would be too challenging. There is also a recent core outcome set for clinical trials for pediatric chronic pain which sheds light on important outcomes including pain severity, pain interference, overall well‐being and adverse events to be measured in all studies.^27^ Youth also recommended having mental health resources available to the study participants after they fill out research surveys, as questions about depression and anxiety might be triggering for some. The setting of outcome measurement was another important question; when and where would be the best time to ask the participants to fill out the outcome measures; during their appointments in the clinic? or a day before or after the appointment at home? There was no consensus among the youth on the best time to complete outcome measures. Responses were divided, with half suggesting that they would prefer to do it in the clinic because they are already there for their visit and the other half said that they would prefer to do it at home to reduce the time spent at the clinic. Three of the youth with chronic pain who participated in this study expressed interest in further working with the CAN‐CHA trial. These youth continue to serve as C4T research advisors and contribute to decision making on the steering committee including finalizing the study protocol, co‐creating study documents including recruitment materials, and a knowledge translation plan to disseminate the study findings.

#### 3.4.2 Study 2: A clinical trial looking at non‐IV treatment for migraine attacks in the emergency department

For the non‐IV treatment for migraine attacks in the emergency department study, the youth voted on which would be their preferred non‐IV intervention in this setting, after having been presented with details on four intervention options. Consensus was reached with the majority of the youth indicating preference for a particular neuromodulation device over the other non‐IV interventions presented as options. With regard to outcome assessment in the emergency department, the youth had mixed preferences for self‐reporting or having the research assistant collect outcomes verbally from the youth. The youth also had mixed preferences for reporting data using paper versus electronic reporting. The youth provided feedback on how to best engage with potential participants in the emergency department setting, including having one‐page summary handouts to give to potential participants, with information about the study interventions and preliminary data. Overall, the youth also unanimously reported interest and support for the concept of testing a non‐IV intervention for acute attacks of migraine in the emergency department.

### 3.5 Youth experience and recommendations for youth engagement

Overall, the feedback obtained participants who completed the modified PPEET was very positive. Regarding communication, all youth (*n* = 8/8 youth who completed the PPEET) agreed or strongly agreed that they had a clear understanding of the focus group purpose, that the supports (information) needed to participate were available and that they had enough information to contribute to the topic being discussed. When asked an open‐ended question about how their participation was supported, one youth responded that “The discussion questions were open ended and allowed for good ideas to be exchanged. I liked how the presenters helped prompt some questions as well.”

With respect to sharing their perspectives, all youth (*n* = 8/8) agreed or strongly agreed that they would share their views freely, 7/8 responded that that their views were heard, that there was a wide range of views discussed, and that individuals represented a broad range of perspectives. One participant elaborated about their experience, “Having chronic intractable migraine for 3.5 years has allowed me to gain a lot of knowledge about chronic migraines. I have trialed through several treatments and these experiences allowed me to share my views towards the research studies, from a patient's perspective”.

When evaluating the impact of the engagement initiative, all youth (*n* = 8) agreed or strongly agreed that the focus groups achieved their stated objectives, were confident that their input would be used by the research teams to design pain studies, and will make a difference to their work (*n* = 8/8). Overall, the youth agreed that this engagement initiative was a good use of their time. Open‐ended responses revealed that youth found visuals like images and graphs to be helpful during the discussion. One youth described their experiences as “It was a respectful environment and I felt like I could share my opinion without judgment even if others felt a different way.”

There were a few areas for improvement in the engagement experience that the youth highlighted after their focus group participation. Specifically, there was a need for engaging a more a gender‐diverse group. One response suggested “There was a gender imbalance (a lot more girls)”. One youth suggested creating a Facebook group as a resource where researchers can post the different opportunities for youth who are interested in becoming research advisers. Another participant suggested posting the engagement opportunities on hospitals' Instagram pages as internet resources associated with hospitals are perceived as a reputable source of information. Furthermore, not all youth felt the same about promoting research opportunities on social media. It was brought up by several youth that social media might not be the best place for recruitment into engagement activities, as some ads might seem like a scam and it is difficult to discern what is genuine on social media.

Due to the COVID‐19 pandemic, plans for in‐person engagement events were modified into online discussion groups. As such we asked youth how to better the virtual‐research engagement experience. Suggestions were made by several youth on how to better facilitate a discussion specifically on Zoom. A few youth commented that it was hard to tell when someone is about to speak and they were cut off a few times. One youth suggested that instead of having people speaking out, they could use the raise hand function and the moderator will call each youth in turn for a smoother conversation. Another youth suggested using the breakout room function “that way people can have more time to share and you will get more information. Some people might feel more comfortable”.

## 4 DISCUSSION

There is a need to raise awareness of the importance of engaging youth in research design and in identifying important outcomes for clinical trials. This study demonstrated the immense value of information youth contribute to improving clinical trial designs. More efficient and meaningful clinical trials will hopefully translate into better pain and headache management strategies for youth moving forward. Our methods provide a model of effective engagement with youth to others as was demonstrated during the evaluation process.

The valuable feedback we received led directly to trial modifications (see Appendix C for a summary table). To address the feedback from the youth, the research team have committed to working with the study site investigators and treating physicians on a recruitment plan that will focus on sending out information in a personalized email to potential participants. The research team will also work on creating an infographic and short video to explain the research study details to potential participants and their parents. Sites will hire research study nurses and coordinators that will be consistently seeing and phoning the participants throughout the whole study. We will attempt to ensure that study visits are scheduled at the same date and time each month. The research team will have a conversation with clinical trial participants about their preferred evidence‐based strategies for pain management (distraction, numbing cream) and fear management strategies for blood tests. Changes to the CAN‐CHA protocol also introduced plans for the research team member to follow up with participants if they miss several (2 or more) research tasks in a week to check‐in on their wellbeing and offer supportive resources as needed. The research team will ensure that daily reminders to complete the tasks are sent to each participant via text message. This is beneficial to researchers who need to collect routine outcome data and participants who feel as though a member of the team has concern for them. And lastly, study sites will work on having snacks available to participants. The research team added a “yes” or “no” question to the daily diary to reflect on the feedback from the youth about an important outcome: “Did you cancel any events (school/extracurricular/social) today due to headaches”. Different pain rating systems were discussed, including the traffic light method, however, using a numeric scale is the most robust and validated measure for the age group (adolescents).^28^ CAN‐CHA has also engaged youth to develop the recruitment materials and social media strategy for clinical trial information sharing. Broad dissemination may present a good strategy to diversify the population who participate in research and advising opportunities; however, methods to increase diversity need to consider that not all populations have equal access to internet and mobile devices.

Unsurprisingly, where possible, especially with COVID‐19, youth would prefer virtual visits, or if in‐person could be in the evening or on a weekend. This should be considered for research engagement activity planning and accommodated where possible. For the CAN‐CHA trial, and many other clinical trials, this unfortunately is not possible due to restricted clinic hours and the need for blood draws to evaluate drug levels and for safety assessments (pregnancy, drug monitoring, liver and kidney function). For the non‐IV treatment for migraine attacks in the emergency department study, the non‐IV intervention was co‐selected along with youth feedback. We will also incorporate youth feedback into how to best present information to potential study participants (i.e., with one‐page handouts), and how to measure outcomes in the emergency department (i.e., by allowing participants several options for how to report outcomes – i.e., electronic or verbally to the research assistant). There are some challenges to conducting patient engagement in research with adolescents such as the need for parental consent, scheduling to accommodate school and social events, aging out of being “youth advisors”, and navigating limited knowledge of clinical trial processes in the public. The reality is that these will be challenges for all clinical trials to navigate and are best mitigated with input directly from youth. The main messages we heard for researchers were to engage youth with open ears, early on and to demonstrate that you value their input. Researchers need to clearly justify which changes will be implemented and which recommendations are not possible in this study design. For example, the traffic light model youth described for pain, is not an age‐validated outcomes assessment tool, thus is not a robust primary outcome. To be able to meaningfully engage youth in designing clinical trials, research teams require access to funding to fairly compensate patient partners, tools to support best practices for engagement, and examples of meaningful patient engagement in clinical trials.

Although the discussion groups positively influenced how the two studies will be conducted, there are several shortcomings to this methodology. First, there could have been one or several dominant participants leading the discussion and their viewpoints are become the representing opinion of the larger group.^20^ The facilitators tried to mitigate that by encouraging all participants to express their thoughts. Second, having the researchers present might have effected the discussion.^20^ In order to reduce bias, the discussion was led by youth partners‐ VWLT and KD, and we referred to the researchers by their first names to reduce power imbalance. Lastly, inevitably there are going to be biases when researching medical cannabis with children based on past experiences and knowledge. However, in this study cannabis was not discussed other than when the two studies were introduced.

## 5 RECOMMENDATIONS FOR MEANINGFUL RESEARCH

During our focus groups and throughout our engagement process we learned several important lessons about working with youth. It is important to meaningfully engage with youth to avoid “tokenism”, or justifying a checkbox on a funding application or manuscript submission.^14^ There are strategies highlighted in other publications^29^, ^30^, ^31^ for ensuring meaningful and collaborative engagement with youth. Engaging patients and the public in research only to prevent criticism or create the appearance of valuing their perspectives can be mitigated by formalizing protocols for engagement.

We encourage more organizations to fund patient engagement, and patient engagement research, as one of the initial components to clinical trials to encourage earlier and more meaningful engagement. One engagement method includes providing opportunities for youth to get involved in the project in different capacities, “through figurative ‘climbing of the ladder’”.^14^ By providing different levels of participation, there is flexibility for the youth to determine the extent they want to collaborate, for example as a discussion group participant, providing written or oral feedback, or as a steering committee member. Each youth can then choose the mode of participation based on their strengths, abilities, and interest.^14^, ^16^, ^32^ For example, our engagement work included 16 youth, three youth advisors of which have chosen to continue as research advisors throughout the CAN‐CHA trial. They have also contributed to live webinars on engaging youth in research and been invited to collaborate on other projects. Providing paid opportunities for youth to be involved in more than just a consultation phase, but as research ambassadors, can have far reaching benefits for increasing clinical trial capacity and knowledge for the next generation.

## 6 CONCLUSIONS

A total of 16 youth participated in a multi‐stage research study to gather their perspectives on the design of two chronic pain clinical trials. Much of the insight provided by the youth on approaches to recruitment/retention and trial barriers are transferable to clinical trials in other disease areas including recommendations for measuring functional outcomes in clinical trials. The engagement methods described here provide an example framework that can be used by other investigators to increase capacity for youth involvement and to meaningfully support autonomy in youth decision making on research participation.

## FUNDING INFORMATION

This project received funding from a SickKids Foundation Early Career Investigator award (LEK) in partnership with the Canadian Institutes for Health Research.

## APPENDIX A

### RECRUITMENT INFORMATION

#### Seeking youth advisors to help co‐design studies about headaches and migraines

##### About the project

Chronic daily headache (CDH) is a common and debilitating disorder. Children with CDH frequently report many symptoms like altered sleep, body pain, orthostatic dizziness, and nausea (in up to 90% of children who experience migraines). At present for the treatment of migraines, all standard of care emergency department interventions are administered intravenously, which is associated with higher rates of adverse events compared to other treatment routes.

The goal of this project is to increase the influence and input of children and adolescents into (1) the development of clinical research on cannabis health products for the treatment of chronic daily headaches and (2) co‐design an intervention for migraine in the emergency department, specifically regarding which novel treatment should be evaluated next.

As a youth advisor, you would be meeting with other youth advisors across Canada. The team organizing this engagement opportunity include a youth with lived experience with headaches, and researchers and health professionals (pharmacist, doctor, psychologist) from the University of Manitoba and the University of Calgary. Solutions for Kids in Pain (SKIP; www.kidsinpain.ca) is a project partner. Your engagement as a youth advisor is part of a research study so you will be asked to sign a consent form before participating.

##### Opportunity

We are looking for 12–15 youth who are 13–18 years old and who live with chronic headaches and/or migraines. As a youth advisor, you will be asked to participate in two online sessions with other youth advisors. The session will be led by a youth who lives with headaches and some of the research/health professional team members. You will be asked to participate in a discussion and share your ideas by talking or typing.

- ***How much time will it take*?** 3 h (two 90 min online sessions)
- ***Session dates*:**
  ⚬ December 5th 2020, 1:00–2:30 PM CT 1:00–2:30 PM CT (11 AM–12:30 PM PDT, 12–1:30 PM MST, 2–3:30 PM EST, 3–4:30 PM ADT)
  ⚬ December 12th 2020, 1:00–2:30 PM CT (11 AM–12:30 PM PDT, 12–1:30 PM MST, 2–3:30 PM EST, 3–4:30 PM ADT)
- ***What would I be asked to do*?**
  ⚬ Willing and available to participate in two 90 min online sessions using the Zoom platform with up to 14 other youth
  ⚬ Share your ideas and thoughts to questions posed by the project team
  ⚬ Fill out an online survey after the groups are finished
  ⚬ No preparation needed
- ***Is it suitable for a beginner*?** Yes. No experience is needed.
- ***Can it be done from home*?** Yes. You would just need a computer.
- ***Compensation*:** $25/h for 3 h ($75 total)

*Who should I contact if I'm interested or have questions*:

Name: Zina Zaslawski

Email: zina.zaslawski@umanitoba.ca

Role: Project coordinator

*Please contact before December 1, 2020*.

## APPENDIX B

### DAILY HEADACHE DIARY QUESTIONS

1. How many hours did you sleep last night?

2. Did you have anything to eat before taking your first dose of the study medication? Y/N

3. Did you have any headaches today? Y/N

3.1. How many did you have today?

3.2. In total approximately how long did the headaches last today? Please use the following format HH:MM

4. Rate your *baseline/average* pain level today, on a scale from 1 to 10

5. Rate your *highest* pain level today, on a scale from 1 to 10

6. Rate your *lowest* pain level today, on a scale from 1 to 10

7. Did you take any other medications (not included in the monthly medication review) today for any reason? Y/N

7.1. Please list the medication and frequency.

For example, Tylenol‐ 3 tabs.

8. Did you avoid attending any events (school/ extracurricular/social) today due to headaches? Y/N

9. Did you see physiotherapist today? Y/N

10. Did you see a psychologist today? Y/N

11. Did you experience any of the following adverse events today? Y/N

11.1. Nausea, vomiting, diarrhea, drowsiness, fatigue, sleepiness, Seizures or convulsions, Decreased or increased appetite, dizziness, Dry mouth, Irritability, Others (mention)

12. Anything else you would like to share with us about your day? (open text)

## APPENDIX C

### RECRUITMENT INFORMATION

#### The main points of action from the meeting with YPREG in September 2020

**Table:**

What you shared with us	What we plan to do
Prefer the blood draw at the same place, same time with the same nurse	Research team will hire study nurses that will be seeing the participants throughout the whole study Research team will try and ensure that study sites are scheduled at the same date/time each month Blood will be drawn in the clinic (participants will not be sent to a separate blood lab)
Each youth has individual pain management and fear strategies for blood tests	Add to the consent that the nurse and youth will decide together on pain management and fear strategies for blood tests. Research team will train research nurses to have a conversation with the youth about their preferred strategies
Would like to have some snacks available, especially during longer appointments and around lunch time	Study sites will work on having snacks available to participants
Be able to see progress based on the daily diary	Research team will figure out the best way to do this. Possible options: in app chart, weekly email updates or other.
Text notifications to complete daily diary	Research team will figure out the best way to do this. Text will provide link to survey, unless using paper copy.
Personalized call to check in for study retention	Study nurse to follow up with participants if they miss several (2 or more) daily diary entries in a week Study nurse to call participant and check in on their wellbeing a week before each study visit
Mental health support available to participants	We will add to the consent that mental health professionals are a part of the care team for each participant and help will be available if needed
Schedule of events table is confusing	Lines will be added and merge cells removed for clarity
Benefits section should come first (good before the bad) and could include compensation	We will re‐order the sections to put benefits first. We do not feel that compensation is a benefit to participating as there is significant time involved (including daily diaries) for participants. We feel that presenting compensation as a benefit may appear coercive.
18 year old participants	Consent form will be modified so that only those 17 and under need their parents' signature
Include what happens when a doctor wants to withdraw but the youth wants to continue the study	Research team will discuss this and it will be included in the consent form.
Background checks could be done as this is a new product with potential for misuse	Discussions on this topic were mixed and the benefits were weighed against the risks (particularly privacy, legality and trust). The research team felt that since these would not be new patients/families (they have to have had chronic pain for at least 6 months to be eligible) and doctors would have a detailed history that this would not be necessary

#### The main points of action from the meeting with youth with headaches in December 2020

**Table:**

Discussion topic	What you shared with us	What we plan to do
Important outcomes	Intensity and frequency. Participation versus withdrawing from/canceling various activities (school and extracurricular). Rating pain from 1 to 10 is not ideal, suggested the traffic light method.	Research team will add a question to the daily diary “Did you cancel any events (school/extracurricular/social) today due to headaches” Y/N. Zina and research team will look into different pain ratings, including the traffic light method.
Reaching participants for recruitment	Directly contacted by a research team member or physician (can be their own). Personalized email or call. Will not respond to an ad on social media.	Research team will work with the physicians at the study sites on a recruitment plan that will send personalized emails to potential participants.
Barriers to participation	A detailed information sheet or short video to explain the study and its impact. Time constrain for both the youth and parents.	Zina will work with research team and CDH YAC to create an infographic and a short video explaining the study for youth and parents.
Participant retention	Emails and text reminders of appointment Text reminders of daily diary	Research team will work on a way of sending out reminders via text or email.
Ease of participation	If possible, virtual appointments Appointments later in the day	The study will collect samples using special tubes, therefore virtual appointments are not possible.
Daily diary	Daily reminders would be helpful. Data collection everyday for data that are more accurate and a better representation of the pattern of headaches.	Research team will make changes to the protocol to collect daily diary information everyday for the duration of the study (6 months).
Sleep measure	About 50/50 for preference. Disturbance‐ headaches mostly prevent from falling and staying asleep. Hard to know if it was sleep that is impairing function or other factors. Impairment‐ getting good sleep due to medication, gathers better information related to quality of life.	Research team reviewed the feedback and decided to include the impairment outcome scale in the study.
Schedule of events	Will be useful to ask all the scales more than twice for better representation of results. The questions about depression and anxiety can be triggering to some	Research team will make changes to the protocol to collect outcome measures every second month (increase from two collection points to three). In addition, mental health resources will be made available to youth
Appointments/filling out outcome measures	About 50/50 for preference. ~ ½ would prefer to do in clinic, since already there for appointment ~½ would prefer to do it at home, to reduce the time spent at the clinic	Research team will make it possible to youth to fill out measure outcomes at home (during a specific window before or after the appointment) or in clinic.
